# Global longitudinal strain assessment in contrast-enhanced echocardiography in breast cancer patients: a feasibility study

**DOI:** 10.1186/s12947-023-00304-w

**Published:** 2023-04-20

**Authors:** Shichu Liang, Mei Liu, Zhiyue Liu, Xiaorong Zhong, Yupei Qin, Ting Liang, Xi Wang, Zhuoqin Tang, Qian Li, He Huang

**Affiliations:** 1 Department of Cardiology, West China Hospital, Sichuan University, No.37 GuoXue Alley, Chengdu, 610041 China; 2 Department of Head and Neck Oncology Cancer Center, West China Hospital, Sichuan University, Chengdu, 610041 China; 3 Clinical Research Center for Breast, West China Hospital, Sichuan University, Chengdu, 610041 China

**Keywords:** Breast cancer, Left ventricular global longitudinal strain, Two-dimensional speckle-tracking echocardiography, Contrast-enhanced echocardiography, Autostrain

## Abstract

**Background:**

Left ventricular global longitudinal strain (GLS) obtained from two-dimensional speckle-tracking echocardiography (2D-STE) can reflect cancer therapy-related cardiac dysfunction in breast cancer (BC) patients, however, the accuracy and reproducibility of 2D-STE are restricted due to poor image quality.

**Methods:**

Between January 2019 and October 2021, 160 consecutive BC patients aged ≥ 18 years were recruited. The 160 BC patients (mean age: 48.41 ± 9.93 years, 100% women) underwent both 2D-STE and Contrast-enhanced echocardiography (CEcho), 125 of whom were included in the measurement of GLS. The intraclass correlation coefficient (ICC) was used to determine the intra- and inter-observer reproducibility of 2D-STE and CEcho-STE. Correlation (r) was calculated using Pearson correlation. Statistical significance was set at *P* < 0.05.

**Results:**

Among 160 BC patients, more segments were recognized by CEcho-STE than by 2D-STE (2,771, 99.53% vs. 2,440, 84.72%). The left ventricular ejection fraction (LVEF) obtained by 2D was lower than CEcho (61.75 ± 6.59% vs. 64.14 ± 5.97%, *P* < 0.0001). The GLS obtained by 2D-STE was lower than CEcho-STE (-21.74 ± 2.77% vs. -26.79 ± 4.30%, *P* = 0.001). The ICC of the intraobserver and interobserver agreements in the CEcho-STE group was lower than that in the 2D-STE group. GLS measurements were in good agreement between the 2D-STE and CEcho-STE groups (*r* = 0.773).

**Conclusions:**

CEcho can overcome some imaging limitations and recognize more segments than 2D, which may provide an LVEF and GLS closer to the true value. Based on AutoStrain, CEcho-STE may serve as a complementary method for those with poor image quality.

**Graphical Abstract:**

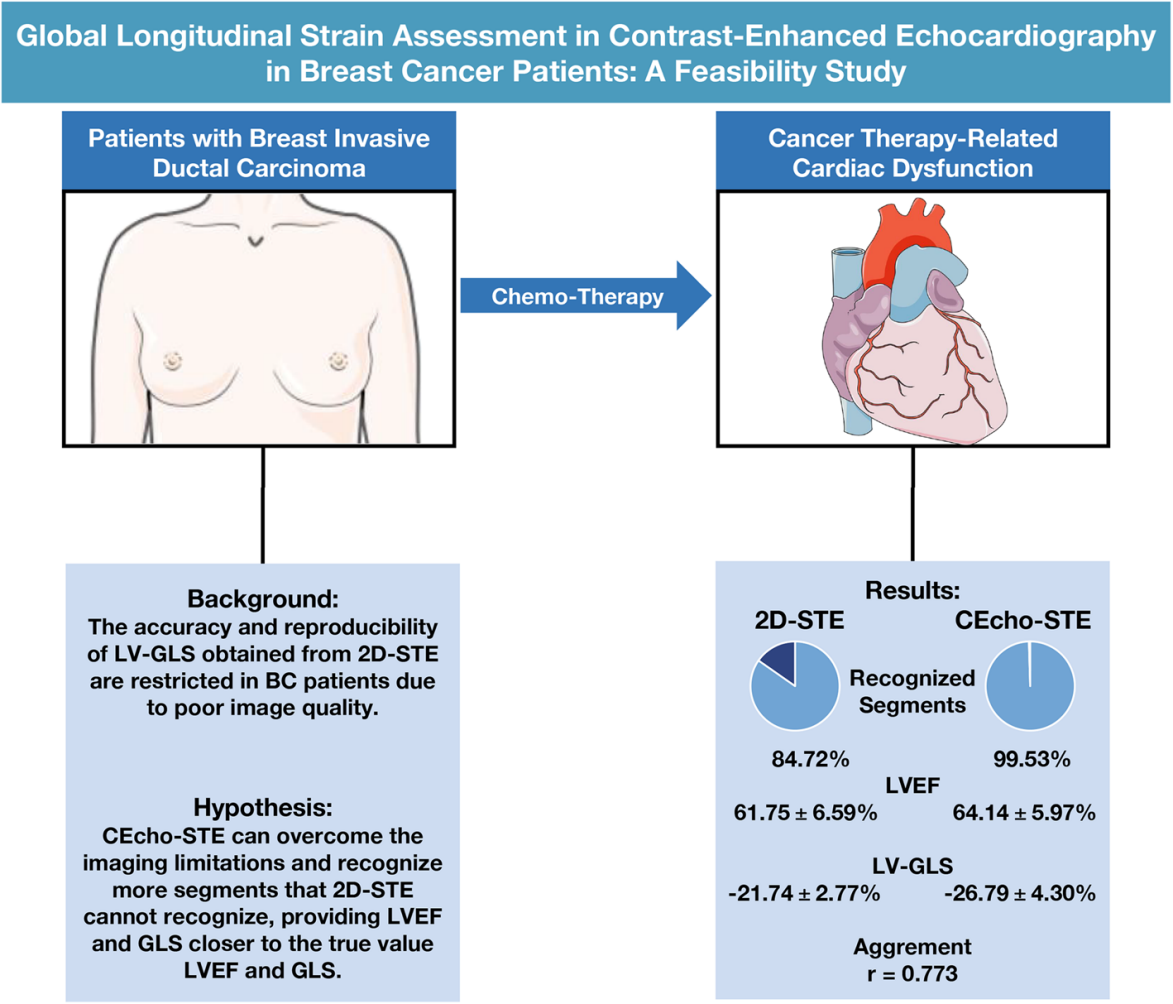

**Supplementary Information:**

The online version contains supplementary material available at 10.1186/s12947-023-00304-w.

## Introduction

Anthracyclines are the core drugs for breast cancer (BC) [1]. Cancer therapy-related cardiac dysfunction (CTRCD), such as cardiomyopathy, heart failure, and pericardial and valvular disease, drastically impacts BC patients’ quality of life and survival [2]. The incidence rate of clinically overt cardiotoxicity is 6%, whereas that subclinical cardiotoxicity is 18% [3]. Therefore, early detection of CTRCD allows timely initiation of cardioprotective therapies, which are vital for maintaining heart function and the entire course of chemotherapy.

Left ventricular (LV) global longitudinal strain (GLS), acquired using two-dimensional speckle-tracking echocardiography (2D-STE), is a sensitive clinical index for detecting early subclinical ventricular dysfunction [4] and has incremental diagnostic and prognostic value [5, 6]. Moreover, GLS contributes to risk classification, a component of CTRCD management [7, 8]. Nevertheless, a significant fraction of BC patients undergo thoracic surgery or radiation therapy, leading to poor quality images, and the endocardial borders do not develop well [9], thus restricting the accuracy and reproducibility of 2D-STE. Poor imaging in BC patients makes STE analysis difficult. Contrast-enhanced echocardiography (CEcho) permits the enhanced detection of microbubbles within the LV cavity and myocardium and is helpful for endocardial border recognition [10]. GLS measurement under contrast conditions is problematic because speckle tracking interferes with microbubbles in the myocardium. However, previous studies have found it feasible, albeit with large intraobserver and interobserver variability [11–14]. AutoStrain is an automated strain analytical software that is co-supported by auto view recognition, auto contour placement, and speckle-tracking technologies. GLS assessment based on AutoStrain can reduce measurement variability [15]. Therefore, it evoked us to try and combine 2D-STE with CEcho, to explore the feasibility and reproducibility of the combination of 2D-STE and CEcho. With the inception of automated methods, we speculated that combining GLS analysis with CEcho could improve GLS analysis.

## Material and methods

### Study design and population

The current research comprised a cross-sectional mixed methodology that combined qualitative and quantitative studies from a registered study (http://www.chictr.org.cn Identifier: ChiCTR1900022108). Consecutive patients with invasive breast ductal carcinoma (including initial treatment and retreatment BC patients) aged ≥ 18 years at the West China Hospital, Sichuan University, were recruited from January 2019 to October 2021. Patients were diagnosed with BC using Version 3. 2018 National Comprehensive CancerNetwork (NCCN) Clinical Practice Guidelines [16].

Exclusion criteria included: (1) severe arrhythmia such as ventricular premature bigeminy, atrial flutter, and atrial fibrillation; (2) cardiac valve disease such as moderate-severe aortic stenosis, aortic regurgitation, mitral stenosis, and mitral regurgitation; (3) myocardial infarction and symptomatic heart failure; (4) a history of cardiac surgery; and (5) renal failure, hypertension, and severe organ injury.

Demographic data, such as age, sex, height, weight, and body surface area (BSA) at baseline, were collected. BSA was calculated using the following formula:

$$\mathrm{BSA }\left({\mathrm{m}}^{2}\right) =0.0061 \times \mathrm{height }\left(\mathrm{cm}\right) +0.0128 \times \mathrm{weight }\left(\mathrm{kg}\right) -0.1529$$

Body mass index (BMI, kg/m^2^) = weight (kg)/height^2^ (m^2^), obesity was defined as BMI ≥ 28 kg/m^2^, and overweight was defined as 24 kg/m^2^ ≤ BMI < 28 kg/m^2^ [17].

All data were collected following approval from the ethics committee of West China Hospital, Sichuan University (Approval Number:20180517). Chemoimmunotherapy and radiotherapy regimens followed the Chinese Society of Clinical Oncology (CSCO) guidelines [18].

### Two-dimensional echocardiography

All patients underwent echocardiographic examinations using an ultrasound system (EPIQ7C; Philips Medical Systems NA, Bothell, WA, USA) via an S5-1 probe (1–5 MHz). All measurements were performed during sinus rhythm according to the American Society of Echocardiography guidelines [19, 20]. Five consecutive cardiac cycles were acquired from each view and digitally stored.

In a parasternal long-axis plane, the left ventricular internal diameter (LVID), left atrial diameter (LAD), right ventricular diameter (RVD), interventricular septal thickness (IVS), and left ventricular posterior wall thickness (LVPW) were measured. Right atrial diameter (RAD) was measured in the apical four-chamber view. Left ventricular mass (LVM) and left ventricular mass index (LVMi) were calculated using the following formulas:

$$\mathrm{LVM (g) }= 0.8 \times 1.04 \times [(\mathrm{LVID }+\mathrm{ LVPW }+\mathrm{IVS})^3-(\mathrm{LVID})^3] + 0.6$$

$$\mathrm{LVMi (\mathrm g/\mathrm m^2)}=\mathrm{LVM (g)}/\mathrm{BSA (m^2)}$$

The mitral inflow velocity was acquired from the apical four-chamber view, and the transmitral peak early diastolic velocity (E) and peak late diastolic velocity (A) were measured. The velocity of the mitral annulus at early diastole (e’) was measured using tissue Doppler imaging, and the lateral (e’ Lat) and septal (e’ Sept) of the mitral valve annulus were recorded. The E/e ratio was used as an index of LV diastolic function. E/e’ ratio = 2 × E / (e’ Lat + e’ Sept).

To minimize respiratory influences, the participants were instructed to hold their breath at the end of exhalation. Proper care was taken to avoid apical foreshortening and to maximize the length from base to apex, and the frame rate was kept at 60–80 frames/s. The endocardium of the left ventricle is displayed. Two-dimensional apical two-, three-, and four-chamber views for five cycles were acquired and stored for later analysis. Left ventricular ejection fraction (LVEF) was measured using modified biplane Simpson’s rule.

### Contrast-enhanced echocardiography

The CEcho study was performed in all patients using amplitude modulation and pulse inversion sequencing (fundamental and harmonic) on an EPIQ7C ultrasound system (EPIQ7C; Philips Medical Systems NA, Bothell, WA, USA) via an S5-1 probe (Frequency of < 2.0 MHz) [20]. Imaging was performed in the apical two-, three-, and four-chamber views with a mechanical index of 0.16 to 0.17, a frame rate of 25 to 30 Hz, and intermittent bolus infusion of lipid-encapsulated sulfur hexafluoride microbubbles SonoVue contrast agent (Bracco SpA, Milan, Italy). A total of 59 mg of SonoVue was diluted with 5 mL of 0.9% saline. Then 0.5 mL of the solution was injected intravenously at a rate of approximately 0.5 mL/min, followed by flushing the intravenous line with 5–10 mL saline, and the process was repeated if necessary. To maintain a stable steady-state concentration of microbubbles and minimize potential measurement bias, images for perfusion analysis were captured within the first 2 min after contrast infusion, with a constant penetration depth, while paying particular attention to the posterior acoustic shadow generated by the contrast, maintaining it at the height of the mitral valve. After optimal left ventricular opacification (LVO), apical two-, three-, and four-chamber views were acquired and stored digitally in a raw format for analysis. LV end-diastolic volume (LVEDV), LV end-systolic volume (LVESV), and LVEF were calculated using the modified biplane Simpson’s rule.

### Measurements of GLS

The unenhanced and contrast-enhanced strains were analyzed offline using fully automated strain analytical software (AutoStrain, Philips) in each patient. Endocardial border tracings were automatically performed on three apical images at end-diastole and end-systole. Manual adjustments were made for images with inadequate endocardial boundary tracing. The contouring was verified using apical images and modified as necessary to ensure optimal endocardial tracking. Good tracking is defined as more than four of the six myocardial segments that can be obtained in all three apical views [19], and GLS measurement was avoided if more than two segments in any one view were not effectively tracked [21]. Poorly tracked segments after necessary manual adjustments were excluded from the GLS analysis. All patients underwent 2D-STE and CEcho-STE (CEcho combined Autostrain) with necessary manual adjustments.

### Image quality scoring

The quality of the images was scored as follows: 3 (> 95% myocardial wall visualization), 2 (70–95% of the relevant wall structures were visualized), 1 (< 70% wall visibility), and 0 (images without clear endocardial border delineation or absent views), according to a previous study [22].

### Intraobserver and interobserver reproducibility

The intraclass correlation coefficient (ICC) was used to determine the intra-, and inter-observer reproducibility of GLS in both groups of 30 randomly selected BC patients. ICC was defined as poor, moderate, and high consistency when it was less than 0.75, between 0.75 and 0.90, and greater than 0.90, respectively [23]. Intra- and inter-observer reproducibility was described as the absolute difference between repeated measurements as a percentage of their means. Intra-observer variability between the first and second measurements (after 30 days) was calculated by the same investigator.

Interobserver variability was calculated between the measurements of two independent investigators, and both investigators were blinded to the results. All GLS measurements were performed by the same investigator (M. Liu and Z. Liu). For the correlation (r) between the different techniques, only patients with an excellent image quality score of 3 were included. r was calculated using the Pearson correlation.

### Statistical analysis

SPSS 26 statistical software (SPSS, Chicago, IL) was used for data processing. Continuous variables with normal distributions were presented as mean ± standard deviation (SD). Independent sample t-tests were used to compare two groups. Continuous variables with skewed distributions are presented as median and interquartile range (25^th^–75^th^ percentile). Intergroup analyses were performed using non-parametric tests (Mann–Whitney U test). Regarding categorical variables (presented as numbers and percentages), the chi-square test (Pearson’s chi-square test) was used to compare proportions between groups. Continuous correction chi-square test was used when the minimum desire frequency was < 5, whereas the Fisher exact probability method was used when the minimum desire frequency was < 1. Linear regression analysis was used to correlate measurements among different techniques, and agreement was assessed using ICC. Statistical significance was set at *P* < 0.05.

## Results

### Study population

**Fig. 1:**
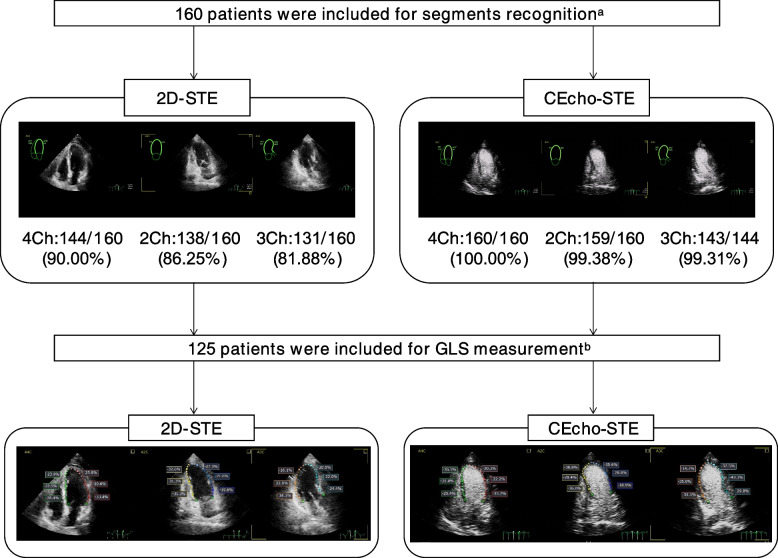
The flow chart of the whole study (**a** good tracking can be obtained in 4 ≥ 6 myocardial segments is considered as good segments recognition; **b** good tracking can be obtained in all apical views is considered as good GLS measurement)

A total of 160 patients were enrolled in the study for segment recognition. The demographic characteristics, treatment-related characteristics, and standard echocardiographic data of all patients at baseline are summarized in Table 1. After segment recognition, 16 patients were excluded because of poor image quality and lack of an apical three-chamber view, and 19 patients were excluded because more than two segments in any one view were not adequately tracked. Therefore, 125 BC patients were enrolled in the GLS measurement. The flowchart of the whole study is displayed in Fig. 1.

**Table 1:** Clinical and echocardiographic characteristics

	Value
**Demographic and clinical characteristics**	
Age, y	48.41 ± 9.93
Sex, female	160 (100%)
Height, cm	157.40 ± 4.67
Weight, kg	59.19 ± 8.43
BSA, m^2^	1.56 ± 0.12
BMI, kg/m^2^	23.91 ± 3.41
Overweight (%)	63 (39.38%)
Obesity (%)	13 (8.13%)
Systolic blood pressure, mmHg	121.77 ± 13.03
Diastolic blood pressure, mmHg	76.81 ± 9.81
Never smoking (%)	160 (100%)
Diabetes mellitus (%)	2 (1.25%)
Hypertension (%)	25 (15.63%)
**Treatment-related characteristics**	
Radiotherapy (%)	100 (62.50%)
Endocrine treatment (%)	46 (28,75%)
Targeted therapy (%)	67 (41.88%)
Oral chemotherapy drugs (%)	14 (8.75%)
Left breast expanders (%)	4 (2.50%)
**Echocardiographic characteristics**	
LVID, mm	44.36 ± 4.49
RVD, mm	20.25 ± 2.41
LAD, mm	30.13 ± 4.72
RAD, mm	32.80 ± 4.58
IVS, mm	8.97 ± 1.74
LVPW, mm	8.15 ± 1.31
LVMi, g/m^2^	75.53 (65.93, 89.49)
MV E, m/s	0.77 ± 0.17
MV A, m/s	0.77 ± 0.21
e' Sept, cm/s	8.48 ± 2.63
e' Lat, cm/s	10.89 ± 2.84
E/e' ratio	8.35 ± 2.30

Data were expressed as: mean ± SD, the median and interquartile interval (first quartile; third quartile) or as a number (percentage)

*LVID* Left ventricular internal diameter, *RVD* Right ventricular diameter, *LAD* Left atrial diameter, *RAD* Right atrial diameter, *IVS* Interventricular septal thickness, *LVPW* Left ventricular posterior wall thickness, *LVMi* Left ventricular mass index, *MV E* Mitral valve peak early diastolic velocity, *MV A* Peak late diastolic velocity, *e' Sept* Septal peak early velocity, *e' Lat* Lateral peak early velocity

### Strain measurements

**Fig. 2:**
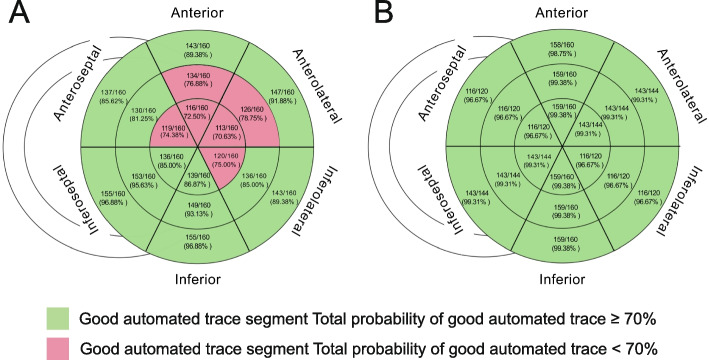
The bull-eye plot of the tracking quality in each segment (A:2D-STE; B:CEcho-STE)

The strain was analyzed using interpretable echocardiographic images. In the included 160 BC patients, 2,440 (84.72%) segments were recognized by 2D-STE, whereas CEcho-STE recognized 2,771 (99.53%) segments. Figure 2 depicts a bull-eye map of the tracking-quality results for each segment. The image score was significantly lower in the 2D-STE group (2.36 ± 0.83 vs. 2.98 ± 0.10, *P* = 0.005). Recognition of the middle and apical segments of the anterior and anterolateral walls and apical segments of the anteroseptal and inferolateral walls in the 2D-STE group were not good.

**Fig. 3:**
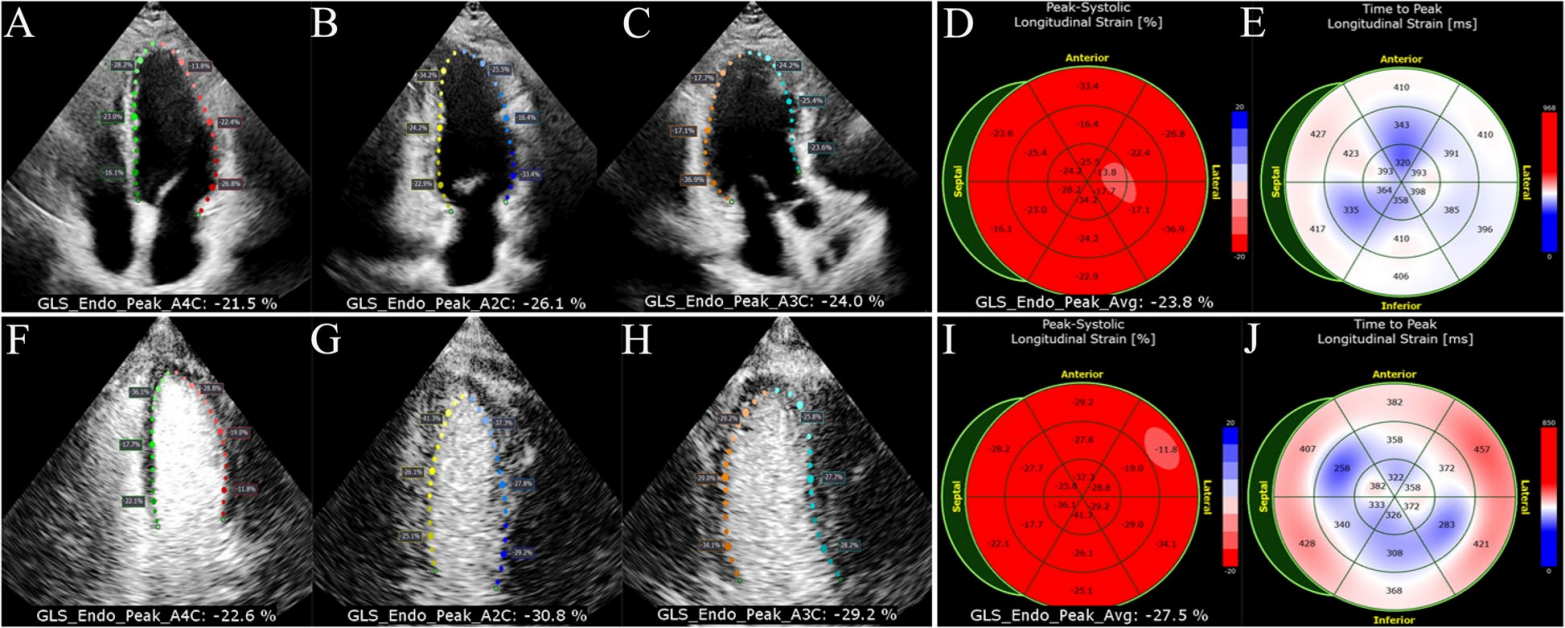
The measurement of LV GLS by AutoStrain (A-E:2D-STE; F-J:CEcho-STE)

The LVEF obtained by 2D was lower than CEcho (61.75 ± 6.59% vs. 64.14 ± 5.97%, *P* < 0.0001). The measurement of GLS via 2D-STE and CEcho-STE is illustrated in Fig. 3 and Supplemental Video 1 and 2. The GLS obtained by the 2D-STE group was lower than CEcho-STE (-21.74 ± 2.77% vs. -26.79 ± 4.30%, *P* = 0.001). The echocardiographic characteristics of 2D-STE and CEcho-STE are displayed in Table 2.

**Table 2:** Cardiac Function and Image Score Between the 2D-STE Group and CEcho-STE Group

	**2D-STE Group**	**CEcho-STE Group**	***P*-value**
**Image Score**	2.36 ± 0.83	2.98 ± 0.10	0.005
**LVEF, %**	61.75 ± 6.59	64.14 ± 5.97	0.000
**GLS, %**^**a**^	-21.74 ± 2.77	-26.79 ± 4.30	0.001

Data were expressed as mean ± SD

^a^only in 125 BC patients

### Intraobserver and interobserver variability

**Fig. 4:**
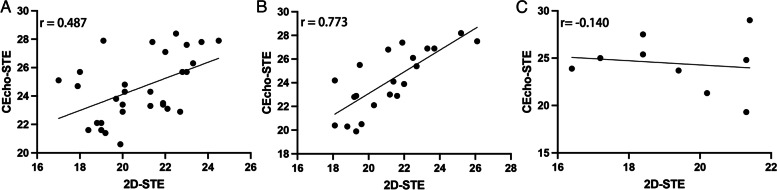
Correlation of GLS Between 2D-STE Group and CEcho-STE Group

As presented in Table 3, there was reasonable interobserver and intraobserver agreement for GLS obtained from the 2D-STE and CEcho-STE groups. However, a slightly worse ICC was observed in the CEcho-STE group. Of the 30 randomly selected patients, 22 had good image quality, while eight had poor image quality. The r was 0.487 for the overall population, 0.773 for the population with good image quality, and -0.140 for the population with poor image quality (Fig. 4).

**Table 3:** Intraobserver and Interobserver Variability Between the 2D-STE Group and CEcho-STE Group

	**2D-STE**		**CEcho-STE**	
	**AD**	**%**	**AD**	**%**
**Intraobserver variability**	2.16	10.41	2.55	10.45
**ICC**	0.895 (95%CI: 0.792–0.948)		0.881 (95%CI: 0.767–0.941)	
**Interobserver variability**	1.98	9..50	2.31	9.40
**ICC**	0.906 (95%CI: 0.814–0.954)		0.808 (95%CI: 0.637–0.903)	

*AD* Absolute difference, *%* coefficient of variation

## Discussion

Abnormal GLS is prevalent in asymptomatic patients exposed to anthracycline chemotherapy, and improved GLS is associated with higher physical activity levels [24, 25]. Therefore, GLS detection is recommended by the ESC Committee during follow-up of CTRCD treatment [26]. In clinical practice, it is challenging to outline endocardial borders due to poor image quality in some patients, which may affect subsequent treatment decisions. As reported, inadequate endocardial visualization can be seen in nearly 20% to 29% of patients with malignancy [27–29]. BC patients are more likely to have poor image quality, mainly due to the interference of breast prostheses and pulmonary gas and the loose fit of the ultrasound probe and chest wall skin. This study excluded 35 (21.88%) BC patients due to poor image quality.

To the best of our knowledge, this is the first study to evaluate the feasibility and reproducibility of GLS assessment under contrast conditions in BC patients. In our study, the GLS measurements were based on 2D-STE and CEcho-STE. We observed that (1) more segments could be recognized by the CEcho-STE group than the 2D-STE group; (2) the absolute values of LVEF and GLS obtained via CEcho-STE were more significant than those in the 2D-STE group; and (3) both 2D-STE and CEcho-STE groups demonstrated good intraobserver and interobserver agreement, although the ICC of the intraobserver and interobserver agreement in the CEcho-STE group was slightly lower.

### CEcho-STE recognizes more segments and larger GLS

CEcho yields a higher left ventricular endocardial border definition [30]. In this study, 2,440 (84.72%) segments were recognized by 2D-STE, whereas CEcho-STE recognized 2,771 (99.53%) segments, respectively. The ratios were 90.0% vs. 100.00% in the apical four-chamber view, 86.25% vs. 99.31% in the apical two-chamber view, 81.88% vs. 99.31% in the apical three-chamber view, which is in agreement with previous findings [31, 32]. Furthermore, the unrecognized segments in the 2D-STE group as middle and apical segments of the anterior and anterolateral walls and apical segments of the anteroseptal and inferolateral walls were impresentable.

We found that LVEF in the CEcho group was more significant than in the 2D group, which aligns with previous studies [8, 33]. Although the study population included BC patients at primary diagnosis and on follow-up, the GLS (-21.74 ± 2.77%) in the 2D-STE group was within the normal range. Interestingly, GLS in the CEcho-STE group (-26.79 ± 4.30%) was more considerable. Previous studies have also observed a higher GLS under contrast conditions [8, 12]. Huqi et al. found a tendency for an increase in the mean GLS with the administration of contrast agents (-13.4 ± 5.8% in the 2D-STE group and -15.3 ± 4.64% in the CEcho-STE, *P* = 0.056) [8]. Furthermore, the mean GLS only significantly differed between the 2D-STE group in patients with poor image qualities (-13.2 ± 3% in the 2D-STE group vs. -15.8 ± 4.5% in the CEcho-STE, *P* = 0.02). Zoppellaro et al. observed higher GLS in the CEcho-STE than in the 2D-STE group (-22.8 ± 5.4% vs. -18.8 ± 4.5%, *P* < 0.001), speculating that the higher GLS may be partly due to the incomplete endocardial border definition when tracking could be more inside in the myocardium [12]. It is worth mentioning that the GLS in our study was higher than that reported in other studies [8, 12], which could be due to the different study populations. LVEF in most of our patients was > 50%. However, the patients included were with heart failure (44% of patients with an LVEF < 50%) [8] and ischemic heart disease (10% of patients with an LVEF < 50%) [12], respectively. Moreover, all patients in our study were female, with a higher GLS than men [34]. The clinical implication of higher GLS in CEcho-STE requires further investigation.

### CEcho-STE causes higher variability

We found that intraobserver and interobserver variability was acceptable in both groups, which is consistent with previous studies [8–14]. However, the variability of the CEcho-STE group was larger than that of the 2D-STE group, and the intraobserver and interobserver correlations were lower than those in the 2D-STE group. Myocardial contrast enhancement may interfere with speckle-tracking analysis; therefore, manual adjustment is required. A semi-automated analysis was adopted in this study. Manual correction was responsible for a significant portion of the variability. Medvedofsky et al. [13] found that speckle-tracking analysis in the apical three-chamber view was challenging because of LVO. Most images acquired from CEcho required manual modifications. This might be because the mechanical index in Medvedofsky et al.’s study [13] was higher than normal (0.1–0.3). As the GLS low mechanical index contrast-specific techniques can provide excellent contrast for endocardial delineation compared with those having high mechanical index [35], we adjusted the mechanical index from 0.16 to 0.17 for fewer microbubbles destruction and better wall motion assessment.

Our study selected only patients with good image quality to evaluate correlations between the two techniques. The poor image quality might cause poor tracking of the myocardial movement, thus causing “unreal” GLS. Although the GLS was manually adjusted, the clinical significance might be better. Kawakami et al. indicated that semi-automated GLS correlates best with LV function. Future studies should strike a balance between measurement variability and clinical significance [15].

### Limitations

Our study has some limitations. Initially, no cardiac magnetic resonance imaging was performed in our study, which means that a potential reference standard might have been missing. Fortunately, we have sufficient good-quality images to make 2D-STE a gold standard to verify the accuracy of CEcho-STE. We believe that the GLS derived from CEcho-STE agrees well with previous studies [8, 13, 14]. Moreover, despite the acceptable intraobserver and interobserver variability in the CEcho-STE group, further studies should explore ways to minimize this variability. This study was only a feasibility study in a single center; for a larger sample size, patients at primary diagnosis and follow-up were enrolled. Despite these limitations, this study may make meaningful contributions to the accurate diagnosis of CTRCD.

## Conclusion

CEcho-STE can overcome some imaging limitations and recognize more segments than 2D-STE, which may provide LVEF and GLS closer to the true value. CEcho-STE may serve as a complementary method in patients with poor image quality. However, more clinical practice is required in the future.

## Supplementary Information

**Additional file 1. **

**Additional file 2.**

## Data Availability

The datasets during and/or analyzed during the current study are available from the corresponding author on reasonable request.

## Abbreviations

BC: Breast cancer
CTRCD: Cancer therapy-related cardiac dysfunction
LV: Left ventricular
GLS: Global longitudinal strain
2D-STE: Two-dimensional speckle-tracking echocardiography
CEcho: Contrast-enhanced echocardiography
NCCN: National comprehensive cancernetwork
BSA: Body surface area
LVID: Left ventricular internal diameter
RVD: Right ventricular diameter
LAD: Left atrial diameter
RAD: Right atrial diameter
IVS: Interventricular septal thickness
LVPW: Left ventricular posterior wall thickness
LVMi: Left ventricular mass index
MV E: Mitral valve peak early diastolic velocity
MV A: Peak late diastolic velocity
e' Sept: Septal peak early velocity
e' Lat: Lateral peak early velocity
LVO: Left ventricular opacification
LVEDV: LV end-diastolic volume
LVEF: Left ventricular ejection fraction
LVESV: LV end-systolic volume
ICC: Intraclass correlation coefficients
